# Mephedrone and Alcohol Interactions in Humans

**DOI:** 10.3389/fphar.2019.01588

**Published:** 2020-01-28

**Authors:** Esther Papaseit, Clara Pérez-Mañá, Elizabeth B. de Sousa Fernandes Perna, Eulalia Olesti, Julian Mateus, Kim PC Kuypers, Eef L. Theunissen, Francina Fonseca, Marta Torrens, Jan G. Ramaekers, Rafael de la Torre, Magí Farré

**Affiliations:** ^1^ Department of Clinical Pharmacology, Hospital Universitari Germans Trias i Pujol (IGTP), Badalona, Spain; ^2^ Department of Pharmacology, Therapeutics and Toxicology and Department of Psychiatry and Forensic Medicine, Universitat Autònoma de Barcelona (UAB), Cerdanyola del Vallès, Spain; ^3^ Department of Neuropsychology and Psychopharmacology, Faculty of Psychology and Neuroscience, Maastricht University, Maastricht, Netherlands; ^4^ Integrative Pharmacology and Systems Neuroscience Research Group, Neurosciences Research Program, IMIM-Hospital del Mar Medical Research Institute, Parc de Salut Mar, Barcelona, Spain; ^5^ Department of Health and Life Sciences, Universitat Pompeu Fabra (CEXS-UPF), Barcelona, Spain; ^6^ Institut de Neuropsiquiatria i Adiccions, Addiction Unit and IMIM, Barcelona, Spain; ^7^ CIBER de Fisiopatología de la Obesidad y Nutrición (CB06/03), CIBEROBN, Santiago de Compostela, Spain

**Keywords:** 4-methylmethcathinone (mephedrone), new psychoactive substance, alcohol (ethanol), interaction, pharmacologic effects, pharmacokinetics

## Abstract

Mephedrone (4-MMC, mephedrone) is a synthetic cathinone derivative included in the class of new psychoactive substances. It is commonly used simultaneously with alcohol (ethanol). The aim of the present study was to evaluate the interactions on subjective, cardiovascular and hormone effects and pharmacokinetics between mephedrone and alcohol in humans. Eleven male volunteers participated as outpatients in four experimental sessions in a double-blind, randomized, cross-over, and placebo-controlled clinical trial. Participants received a single oral dose of 200 mg of mephedrone plus 0.8 g/kg of alcohol (combination condition); 200 mg of mephedrone plus placebo alcohol (mephedrone condition); placebo mephedrone plus 0.8 g/kg of ethanol (alcohol condition); and placebo mephedrone plus placebo alcohol (placebo condition). Outcome variables included physiological (blood pressure, heart rate, temperature, and pupil diameter), psychomotor (Maddox wing), subjective (visual analogue scales, Addiction Research Center Inventory 49 item short form, and Valoración de los Efectos Subjetivos de Sustancias con Potencial de Abuso questionnaire), and pharmacokinetic parameters (mephedrone and ethanol concentrations). The study was registered in ClinicalTrials.gov, number NCT02294266. The mephedrone and alcohol combination produced an increase in the cardiovascular effects of mephedrone and induced a more intense feeling of euphoria and well-being in comparison to the two drugs alone. Mephedrone reduced the sedative effects produced by alcohol. These results are similar to those obtained when other psychostimulants such as amphetamines and 3,4-methylenedioxymethamphetamine are combined simultaneously with alcohol. The abuse liability of mephedrone combined with alcohol is greater than that induced by mephedrone alone.

## Introduction

During the previous decade, numerous non-conventionally listed psychoactive compounds, or new psychoactive substances (NPS), have emerged on the illicit drug market to replace controlled ones (EMCDDA, 2016). Ease of availability combined with relatively low prices, and high purity compared to classical street drugs, plus popularization *via* Internet-driven social media, have contributed notably to their increasing presence on the drug scene. Of the 670 NPS detected by the European Union’s early warning system, synthetic cathinones make up the second largest group (ACMD, 2010; Papaseit et al., 2014; EMCDDA, 2019).

Mephedrone (4-methylmethcathinone, 4MMC, drone, M-CAT, White Magic, meow meow), also known as “bath salt,” “plant feeder,” and/or “legal high,” has emerged as a prototypical synthetic cathinone surpassing the popularity of other NPS (Vardakou et al., 2011; Bretteville-Jensen et al., 2013). It is a beta-keto amphetamine analogue, structurally and pharmacological related to 3,4-methylenedioxymethamphetamine (MDMA) which it was become its legal alternative (Green et al., 2014; Liechti, 2015). In vitro pharmacological assays have characterized mephedrone as a non-selective releaser and inhibitor of their uptake at the monoamine transporter (Baumann et al., 2012; Simmler et al., 2013; Baumann et al., 2013; Rickli et al., 2015; Saha et al., 2015). Recently, initial data regarding the human pharmacology of mephedrone have confirmed its psychostimulant-like effects which were first reported by recreational users in forums, surveys, and naturalistic and observational studies (Dargan et al., 2010; Winstock et al., 2011; Freeman et al., 2012; Papaseit et al., 2016; Homman et al., 2018). Following controlled oral administration, mephedrone induces cardiovascular-stimulant and euphoric effects with a high abuse liability characterized by earlier onset and shorter duration in comparison to MDMA and other amphetamine derivatives (d-amphetamine, methamphetamine and methylphenidate) (Mayo and de Wit, 2015; Papaseit et al., 2016; Dolder et al., 2017; Dolder et al., 2018).

In spite of its illicit status, recreational use of mephedrone continues to be present on the drug scene (Winstock et al., 2011; Deluca et al., 2012; González et al., 2013; Kelly et al., 2013; Mixmag’s Drug Survey: The Results, 2014; CSEW, 2018). In 2015, data from the European Drug Report estimated a previous year prevalence of 3% among club-goers (EMCDDA, 2015). Based on results from the Crime Survey for England and Wales, previous year use of mephedrone among 16- to 34-year-olds was estimated at 0.2%; down from 0.5% in 2015/2016, and 1.1% in 2014/15 (CSEW, 2018; EMCCDA, 2018). In the United Kingdom, there have also been reports of “slamming”—the intravenous injection of mephedrone and other drugs, such as methamphetamine and gamma hydroxybutyrate, immediately before/during sex in groups of men who have sex with men at “chemsex” parties (Melendez-Torres et al., 2018). Mephedrone injecting has been reported as occurring mainly among individuals who have previously injected other drugs (e.g. heroin users), those who have switched from snorting mephedrone, and among younger users (UNODC, 2017).

In addition, mephedrone is linked to an intensive and repetitive administration pattern in which other drugs are concomitantly consumed (Schifano et al., 2011; Deluca et al., 2012). In these social scenes (e.g., nightclubs, music festivals, rave parties), the use of mephedrone and alcohol is the most common two-drug combination reported among NPS recreational users (EMCDDA, 2010; Carhart-Harris et al., 2011; Winstock et al., 2011). Under these conditions, users often report combining mephedrone with alcohol to either heighten its effects or ameliorate the come-down, a particularly unpleasant experience following mephedrone consumption (Newcombe, 2009; Carhart-Harris et al., 2011; O’Neill and McElrath, 2012).

With respect to acute mephedrone intoxication cases, it is notable that in 18.2% of cases, alcohol was also present (EMCDDA, 2016). Indeed, this concomitant use of mephedrone and alcohol in humans has led to several acute toxicities (McGaw and Kankam, 2010) and fatalities (Maskell et al., 2011; Regan et al., 2011; Cosbey et al., 2013; Elliott and Evans, 2014; Loi et al., 2015; Corkery et al., 2017; Papaseit et al., 2017). The latest results for acute drug toxicity presentations from the European Drug Emergencies Network (Euro-DEN) indicate mephedrone as the most common NPS involved (n = 88). The main acute effects include agitation, anxiety, palpitations and chest pain. Recently, in Poland an increased number of acute intoxications with mephedrone (binge episodes) in combination primarily with alcohol and also other substances have been detected (Ordak et al., 2018).

Despite the potentially added risks of mephedrone combined with alcohol, experimental data in humans about interactions between mephedrone and alcohol are very limited. Experimental studies in animal models concerning the effects induced after single and repeated mephedrone and alcohol administration, demonstrated that alcohol increases stimulant and rewarding effects of mephedrone (Ciudad-Roberts et al., 2015), and can also potentiate the neurotoxic properties of mephedrone in adolescent mice (Ciudad-Roberts et al., 2016). A recent study has concluded that mephedrone in combination with alcohol enhances the psychostimulant effect of mephedrone measured as locomotor activity. Given that both serotonin (5-HT) and dopamine are also related with reward and impulsivity, the observed effects point to an increased risk of abuse liability when combining mephedrone with alcohol compared with the sole administration of these drugs (López-Arnau et al., 2018). A part of the present study, including the neurocognitive performance effects of mephedrone and alcohol, have been formerly published (de Sousa Fernandes Perna et al., 2016). The results showed that whilst alcohol intoxication generally impaired performance, mephedrone improved psychomotor performance, impaired spatial memory but it did not affect divided attention performance. Nevertheless, the stimulatory effects of mephedrone were not enough to compensate for the impairing effects of alcohol on most performance parameters.

The present study was designed to assess the subjective, cardiovascular and hormone effects and pharmacokinetics following the interaction between mephedrone and alcohol under controlled co-administration in humans. The findings presented in this paper are part of the previously mentioned study focusing on the physiological and subjective effects and pharmacokinetics of both substances.

## Materials and Methods

### Subjects

Twelve healthy male subjects were recruited by word of mouth. Eligibility criteria required the recreational use of amphetamines, ecstasy, mephedrone, or cathinones with a lifetime minimum of six times, and on at least two occasions during the year prior to participation, without any serious adverse reaction; recreational use of alcohol (less than four units of alcohol per day) and previous experience of acute alcohol intoxication; and no history of abuse or drug dependence according to the Diagnosis and Statistical Criteria for Mental Disorders IV-R for any other substances except nicotine (in smokers).

All participants completed the study except for one who dropped out due to personal circumstances after participating in three experimental sessions (final n = 11). The participants had a mean age of 28 years (range 22–39 years), mean weight 71.8 kg (range 56.8–83.3 kg), mean height 173.5 cm (range 164.0–180.0 cm) and body mass index 23.8 kg/m^2^ (range 19.0–26.8 kg/m^2^). They had experience with mephedrone (36.4%), MDMA (90.9%), and cocaine (100%). The participants drank an average of 2.4 units of alcohol per day (range 1.0–4.0). All but five were smokers (mean five cigarettes/day, range 2–15).

Prior to their inclusion the participants underwent a general medical examination, including blood laboratory tests, urinalysis, 12-lead electrocardiogram (ECG), and a Psychiatric Research Interview for Substance and Mental Disorders. Participants completed a training session to familiarize themselves with testing procedures and questionnaires. In addition, in order to reduce variability in pharmacokinetics of mephedrone and to avoid the possibility that subject carriers of allelic variants leading to the PM phenotype for CYP2D6 might be at increased risk of acute toxicity, only subjects who were phenotypically CYP2D6 extensive metabolizers were included (de la Torre et al., 2005). The protocol was approved by the local Research Ethics Committee (CEIC-Parc de Salut Mar, Barcelona, Spain). The study was conducted in accordance with the Declaration of Helsinki and Spanish laws concerning clinical trials and registered in ClinicalTrials.gov (number NCT02294266). The volunteers were financially compensated.

Safeguard measures taken to ensure participant welfare while they were participating in the study included health controls before each session (a phone call one day before each session to ensure health status), during sessions (medical examinations at baseline, clinical monitoring with continuous ECG during 10 h and a psychiatric evaluation) and after sessions (medical examination 24 h after each session), a final control including medical examination and blood and urine chemistry were done 3–7 days after the last session, and a final phone call after 3–4 weeks.

### Drugs

Mephedrone was supplied by the Spanish Ministry of *Justice and Ministry of Health*. Mephedrone and placebo capsules were prepared as opaque, white, soft gelatin capsules under the supervision of the Pharmacy Unit of the Hospital del Mar. Acute alcohol intoxication was induced by the ingestion of a beverage containing vodka (Absolut^®^, Ahus, Sweden) diluted in lemon-flavored water (Fontvella^®^) to mask the placebo drink. The alcohol-placebo drink was lemon-flavored water (Fontvella^®^).

### Study Design

The study was double-blind, double-dummy, randomized, cross-over, and placebo-controlled. The four drug conditions consisted of a single oral dose: 200 mg of mephedrone plus 0.8 g/kg of alcohol (combination condition); 200 mg of mephedrone plus placebo alcohol (mephedrone condition); placebo mephedrone plus 0.8 g/kg of alcohol (alcohol condition); and placebo mephedrone plus placebo alcohol (placebo condition). The dose of mephedrone was selected based on previous results of the first study evaluating pharmacological effects of mephedrone in humans under controlled and experimental administration. This dose was selected after a series of pilot studies that included single oral doses of 50, 100, 150, and 200 mg of mephedrone, and 100 mg MDMA, being the dose of 200 mg well tolerated and produced similar effects to MDMA (Papaseit et al., 2016). The dose of alcohol and the combination were selected based in previous psychostimulant drug and alcohol interaction studies (Farré et al., 1993; Hernández-López et al., 2002).

### Experimental Sessions

Subjects were admitted to the Clinical Research Unit facilities at 07:45 a.m. after an overnight fast. Upon arrival, they were questioned about any drug consumption or event that could affect their participation. They had been requested to refrain from using any psychoactive drug for a minimum of seven days prior to the study and throughout it, and from consuming caffeinated products for 24 h and alcohol for 48 h. A urine sample was collected for drug testing (Instant-View^®^, Multipanel 10 Test Drug Screen, Alfa Scientific Designs Inc., Poway, CA-USA). They remained in a calm and comfortable laboratory environment during the entire session. Tobacco smoking was not permitted in the sessions. Last cigarette was allowed 2 h before admission.

At the beginning of each experimental session baseline measures were performed. Mephedrone or matched placebo (one capsule) was administered at 8:30 a.m. in a fasting state with 100 milliliters (ml) of bottled water (Fontvella^®^). Alcohol or matched placebo was administered at 9:00 a.m., 30 min after mephedrone or matched placebo administration. The total volume of the beverage (350 ml) was consumed in 15 min (one-third volume every 5 min).

Four, 6, and 10 h after administration a light breakfast, a meal, and a snack were provided to the participants/subjects, respectively. A psychiatric evaluation was performed 8 h after administration and adverse effects were assessed during each experimental session and the following day.

### Physiological Measures

Non-invasive systolic blood pressure (SBP), diastolic blood pressure (DBP), heart rate (HR), and oral temperature (T) were repeatedly recorded at: -45 min, 0 h (baseline, immediately prior to capsule administration), 0.25, 0.5 (immediately prior to beverage administration), 0.75, 1, 1.5, 2, 3, 4, 6, 8, 10, and 24 h following initial drug administration. All assessments were carried out with a DinamapTM 8100-T vital signs monitor (Critikon, Tampa, Fla., US). Pupil diameter (PD) and the Maddox-wing device were recorded at: 0 h (baseline, immediately prior to capsule administration), 0.25, 0.5 (immediately prior to beverage administration), 0.75, 1, 1.5, 2, 3, 4, 6, 8, 10, and 24 h following initial drug administration. Pupillary diameter was recorded with a Haab pupil gauge and Maddox wing measures were expressed in diopters along the device’s horizontal scale. The Maddox-wing device measures the balance of extraocular muscles and quantifies exophoria as an indicator of extraocular muscle relaxation, and esophoria as an indicator of extraocular muscle tension. For safety reasons ECG was continuously monitored along 10 h using a DinamapTM Plus vital signs monitor (Critikon, Tampa, Fla., US).

### Subjective Effects

Subjective effects were measured using a set of 23 visual analogue scales (VAS), the 49-item Addiction Research Center Inventory short form (ARCI), the Evaluation of Subjective Effects of Substances with Abuse Potential questionnaire (VESSPA-SEE), and a pharmacological class identification questionnaire.

VAS (100 mm) were labeled at opposite ends with different adjectives ranging from “not at all” to “extremely” (Farré et al., 2004; Peiró et al., 2013; Farré et al., 2015; Papaseit et al., 2016). Subjects were asked to rate effects from among “high,” “drunkenness,” “stimulated,” “any effect,” “good effects,” “bad effects,” “liking,” “content,” “drowsiness,” “dizziness,” “confusion,” “fear,” “depression or sadness,” “changes in distances,” “changes in colors,” “changes in shapes,” “changes in lights,” “hallucinations-seeing of lights or spots,” “changes in hearing,” “hallucinations-hearing sounds or voices,” “hallucinations-seeing animals, things, insects or people,” “different or changed unreal body feeling,” and “different or unreal surroundings”.

The Spanish validated version of the short-form ARCI (Lamas et al., 1994), which consists of a true/false 49-item questionnaire, it is a validated instrument for determining subjective drug effects including five subscales: PCAG (pentobarbital-chlorpromazine-alcohol group, a measure of sedation); MBG (morphine-benzedrine group, a measure of euphoria); LSD (lysergic acid diethylamide group, a measure of dysphoria and somatic symptoms); BG (benzedrine group, a stimulant subscale relating to intellectual efficiency and energy); and A (amphetamine, a measure of d-amphetamine effects) (Farré et al., 2015).

The VESSPA-SEE is a questionnaire that measures changes in subjective effects caused by different drugs including MDMA. It includes six subscales: sedation (S), psychosomatic anxiety (ANX), changes in perception (CP), pleasure and sociability (SOC), activity and energy (ACT), and psychotic symptoms (PS) (Poudevida et al., 2003; Papaseit et al., 2016).

The pharmacological class identification questionnaire asks about the class of drugs the participants believed they had been given at each administration (Rush et al., 1995). The options included placebo, benzodiazepine (e.g., valium, diazepam, tranxilium, rophipnol), alcohol, stimulant (amphetamine), designer drugs (ecstasy), cocaine, hallucinogen (e.g., LSD, mescaline), cannabinoids (e.g., marijuana, hashish), ketamine (special K), and gamma hydroxybutyrate (liquid ecstasy).

The VAS were administered at: -45 min (baseline), 0.25, 0.5 (immediately prior to beverage administration), 0.75, 1, 1.5, 2, 3, 4, 6, 8, 10, and 24 h following initial drug administration. ARCI and VESSPA-SEE were administered at: -45 min, 0.25, 0.5 1.5, 2, 3, 4, 6, 8, and 10 h following initial drug administration. The pharmacological class identification questionnaire was given at 8 h following initial drug administration.

### Pharmacokinetics

Blood samples for the determination of mephedrone were collected during each experimental session at -5 min (0 h, baseline), 0.25, 0.5, 0.75, 1, 1.5, 2, 3, 4, 6, 8, 10, and 24 h following drug administration. Urine was collected until 24 h (data not shown). Mephedrone plasma concentrations were quantified by gas chromatography-mass spectrometry (GC-MS). A liquid–liquid extraction was performed with tert-butyl methyl etherm and the silylation reagent [N-methyl-N-(trimethylsilyl) trifluoroacetamide] was used for the derivatization of mephedrone (Papaseit et al., 2016; Olesti et al., 2017).

Blood samples for the determination of alcohol were collected during each experimental session at -5 min (0 h, baseline), 0.75, 1, 1.5, 2, 3, 4, 6, 8, and 10 h after drug administration. Urine was collected until 24 h (data not shown). Alcohol plasma concentrations were determined with an enzymatic test (DRI Ethyl Alcohol Assay, Thermo Scientific) in an autoanalyzer (Indiko Plus, Thermo Fisher Scientific).

Blood samples for the determination of cortisol were collected during each experimental session at -5 min (0 h, baseline), 1, 2, 4, 6, and 8 h following drug administration. They were centrifuged at 3,000 rpm for 10 min at 4°C, plasma and serum were removed and frozen at -220°C until analysis. Cortisol plasma concentrations were determined by fluorescence polarization immunoassay (Abbott Laboratories, Chicago, IL) according to the manufacturer’s instructions (Kobayashi et al., 1979).

### Statistical Analysis

Statistical analysis was performed for eleven participants.

Values from physiological and subjective effects were transformed to differences from baseline. The peak effects in the first 6 h after first drug administration (maximum absolute change from baseline values, E_max_), and the 6-h area under the curve (AUC) of effects versus time were calculated by the trapezoidal rule for each variable.

Peak concentration (Cmax) and time to reach peak concentrations (Tmax) from mephedrone, alcohol and cortisol plasma concentrations were determined using PKSolver, a freely available add-in program for Microsoft Excel (Joel Usansky, Atul Desai, and Diane Tang-Liu, Department of Pharmacokinetics and Drug Metabolism, Allergan, Irvine, CA, USA). Area under the concentration-time curve from mephedrone, alcohol and cortisol (AUC0-6), mephedrone (AUC0-24), and alcohol (AUC0-10) were calculated by the linear trapezoidal rule.

Firstly, these transformations were analyzed using a two-way analysis of variance (ANOVA) test to study the influence of some participant factors as age, body mass index, weight, smoking and alcohol use in the different parameters calculated. Because the results showed only marginal statistically significant results for factors/interactions, the analysis was rejected (11 variables showed significant results for a total number of 200 comparison, percentage 5.5%). Subsequently, the statistical analysis presented was performed without considering those factors.

Then, these transformations were analyzed by means of one-way repeated-measures analysis of variance (ANOVA) with drug condition as factor. In case of significant differences among treatment conditions in ANOVA, post-hoc multiple comparisons were performed using the Tukey test. Time course (T-C) of effects was analyzed employing two-way repeated-measures ANOVA with Treatment condition and Time (0–6 h) as factors. When Treatment condition, or the Treatment condition × Time interaction, were statistically significant, multiple Tukey post-hoc comparisons were performed at each time point. The difference in time to reach peak effects (T_max_) values among conditions was assessed with the nonparametric Friedman test. When significant results among conditions were detected post-hoc multiple comparison was performed applying the Wilcoxon signed rank test adjusting the *p* value to six comparisons (*p* < 0.008).

All statistical tests were performed at each time point using the PASW Statistics 18.0 (SPSS Inc., Chicago, IL, USA). A value of *p* < 0.05 was considered statistically significant.

## Results

### Global Results

**Table 1** shows the summary of physiological and subjective effects where at least one statistical difference (peak, AUC) was found in the ANOVA and multiple comparison post-hoc test analyses. Furthermore, it includes T-C points that presented significant differences in ANOVA and the multiple-comparison post-hoc tests. **Table 2** shows the Peak effect values of variables where at least one statistical difference was found in the ANOVA.

**Table 1:** Summary of statistically significant results of the physiological parameters and subjective effects (n = 11) observed after administration of mephedrone plus alcohol (mephedrone–alcohol), mephedrone plus placebo alcohol (mephedrone), placebo mephedrone plus alcohol (alcohol), and placebo mephedrone plus placebo alcohol (placebo condition).

Variable				Tukey’s Multiple Comparison Test (*p < 0.05; **p < 0.01)					
	ANOVA			Placebo			Mephedrone		Alcohol
	Parameter	F/X^2^	p Value	Alcohol	Mephedrone	Mephedrone–alcohol	Alcohol	Mephedrone–alcohol	Mephedrone–alcohol
**Physiological effects**									
**SBP**	AUC (df = 3,30)	22.107	<0.001	NS	**	**	**	NS	**
	Peak (df = 3,30)	38.667	<0.001	NS	**	**	**	NS	**
	T_max_	11.97	0.007	NS	NS	*	NS	NS	NS
	T-C (df = 3,270)	13.924	<0.001	0.75*,4*	0.5**,0.75**,1**, 1.5**,2**,3**	0.5**,0.75**,1**, 1.5**,2**,3**	0.5**,0.75**,1**, 1.5**,2**,3**,4**	2*	0.5**,0.75**,1**,1.5**,2**,3**,4**
**DBP**	AUC (df = 3,30)	17.087	<0.001	NS	**	**	**	NS	**
	Peak (df = 3,30)	20.138	<0.001	NS	**	**	**	NS	**
	T_max_	9.54	0.023	NS	NS	NS	NS	NS	NS
	T-C (df = 3,270)	5.526	<0.001	6*	0.5*,0.75**,1**,1.5**,2**	0.5**,0.75**,1**, 1.5**,2**	0.5**,0.75**,1**, 1.5**,2**,3*,4**,6**		0.5**,0.75**,1**,1.5**, 2**,3*,4**
**HR**	AUC (df = 3,30)	25.960	<0.001	NS	**	**	NS	**	**
	Peak (df = 3,30)	23.570	<0.001	NS	**	**	**	NS	**
	T_max_	9.38	0.025	NS	*	NS	NS	NS	NS
	T-C (df = 3,270)	10.750	<0.001	0.75**,1**,3*, 4**,6*	0.5**,0.75**,1**, 1.5**,2**,3**,4**, 6*	0.5**,0.75**,1**, 1.5**,2**,3**,4**,6**	0.5**,0.75**,1**, 1.5**,2**,3**	1.5**,2**,3**,4**,6**	0.5*,0.75**,1**, 1.5**, 2**,3**,4**, 6**
**T**	AUC (df = 3,30)	6.475	0.002	NS	**	NS	*	NS	NS
	Peak (df = 3,30)	1.420	0.252						
	T_max_	2.93	0.402						
	T-C (df = 3,270)	4.598	<0.001	0.75**	1.5**,2**,3**,4**, 6**	0.75**,1**,2**,4**,6**	0.75**,1.5**,2**,3**,4**,6**	0.75**,1**,1.5**	0.75**,1**,1.5*,2*,3**,4**
**PD**	AUC (df = 3,30)	20.973	<0.001	NS	**	**	**	NS	**
	Peak (df = 3,30)	25.333	<0.001	NS	**	**	**	NS	**
	T_max_	14.24	0.003	NS	*	NS	NS	NS	NS
	T-C (df = 3,270)	8.972	<0.001		0.5*,0.75**,1**, 1.5**,2*	0.5**,0.75**,1**,1.5**, 2**, 3**	0.5*,0.75**,1**,1.5**,2*	2*,3**	0.5**,0.75**,1**,1.5**,2**,3**
**Maddox-wing**	AUC (df = 3,30)	7.674	0.001	NS	NS	NS	**	NS	*
	Peak (df = 3,30)	13.1320	<0.001	NS	**	NS	**	NS	**
	T_max_	6.21	0.102						
	T-C (df = 3,270)	4.585	<0.001	1**,1.5*,4*	0.75**,1**,1.5**,2*	0.75**,1*	0.5**,0.75**,1**,1.5**,2**,3**,4**	1**, 1.5*	0.75**,1**,1.5**,2**,4**
**Visual Analogue Scales**									
**High**	AUC (df = 3,30)	14.998	<0.001	NS	NS	**	NS	**	**
	Peak (df = 3,30)	27.105	<0.001	NS	**	**	**	NS	**
	T_max_	28.39	<0.001	NS	*	*	NS	*	*
	T-C (df = 3,270)	10.564	<0.001		0.5**,0.75**, 1**,1.5**,2*	0.75**, 1**, 1.5**, 2**,3**, 4**	0.5**, 0.75**, 1**, 1.5**, 2*	0.5*, 0.75**, 1.5**,2**,3**,4*	0.75**, 1**,1.5**,2**,3**,4**
**Drunkenness**	AUC (df = 3,30)	20.076	<0.001	**	NS	**	**	**	NS
	Peak (df = 3,30)	30.600	<0.001	**	NS	**	**	**	NS
	T_max_	30.12	<0.001	*	NS	*	*	*	NS
	T-C (df = 3,270)	8.780	<0.001	0.75**,1**,1.5**,2**,3**		0.75**,1**,1.5**,2**,3*	0.75**,1**,1.5**, 2**,3**	0.75**,1**,1.5**,2**,3*	0.75**,1**,1.5**
**Stimulated**	AUC (df = 3,30)	9.434	<0.001	NS	NS	**	NS	*	*
	Peak (df = 3,30)	17.026	<0.001	*	**	**	NS	NS	**
	T_max_	23.69	<0.001	*	*	*	NS	NS	NS
	T-C (df = 3,270)	5.568	<0.001	0.75**, 1**,1.5**	0.5**,0.75**,1**,1.5**	0.5**,0.75**,1**,1.5**,2**,3**	0.5**,0.75**	1.5**,2**,3**	0.5**,0.75**,1**,1.5**, 2**
**Any effect**	AUC (df = 3,30)	13.702	<0.001	**	NS	**	NS	**	NS
	Peak (df = 3,30)	21.813	<0.001	**	**	**	NS	NS	NS
	T_max_	24.38	<0.001	*	*	*	NS	NS	NS
	T-C (df = 3,270)	6.411	<0.001	0.75**,1**,1.5**,2**	0.5**,0.75**,1**,1.5**	0.5**,0.75**,1**, 1.5**,2**,3**	0.5**	1*,1.5**,2**,3**	0.5**,1.5**,2*
**Good effects**	AUC (df = 3,30)	14.115	<0.001	NS	NS	**	NS	**	**
	Peak (df = 3,30)	22.754	<0.001	NS	**	**	*	NS	**
	T_max_	19.21	<0.001	*	*	*	NS	NS	NS
	T-C (df = 3,270)	6.173	<0.001	1**.1.5*	0.5**,0.75**,1**, 1.5**,2**	0.25**,0.5**,0.75**, 1**,1.5**,2**,3**	0.75**,1*,1.5*	0.25**,0.5**,3**	0.25**,0.5**,0.75**, 1**,1.5**,2*,3**
**Bad effects**	AUC (df = 3,30)	3.426	0.030	*					
	Peak (df = 3,30)	2.384	0.089						
	T_max_	8.29	0.040	NS	NS	NS	NS	NS	NS
	T-C (df = 3,270)	1.164	0.268						
**Liking**	AUC (df = 3,30)	12.629	<0.001	NS	NS	**	NS	*	**
	Peak (df = 3,30)	15.248	<0.001	NS	**	**	*	NS	**
	T_max_	17.67	0.001	NS	*	*	NS	NS	NS
	T-C (df = 3,270)	5.756	<0.001		0.5*,0.75**,1**, 1.5**	0.5**,0.75**,1**, 1.5**,2**,3**	0.5*,0.75**,1**	1.5**,2**,3**	0.5**,0.75**,1**, 1.5**,2**,3**
**Content**	AUC (df = 3,30)	10.094	<0.001	NS	NS	**	NS	**	**
	Peak (df = 3,30)	16.287	<0.001	**	**	**	NS	NS	*
	T_max_	22.4	<0.001	*	*	*	NS	NS	NS
	T-C (df = 3,270)	4.726	<0.001	1**,1.5**,2*	0.75**,1**,1.5**, 2*	0.5**,0.75**,1**, 1.5**,2**,3**	0.75**	1.5**,2**,3**	0.5**,0.75**,1**,1.5**, 2**,3**
**Drowsiness**	AUC (df = 3,30)	6.629	0.001	**	NS	NS	**	NS	*
	Peak (df = 3,30)	5.383	0.004	**	NS	NS	*	NS	NS
	T_max_	14.73	0.002	NS	NS	NS	NS	*	NS
	T-C (df = 3,270)	2.117	<0.001	2*,3**,4**		6*	2**,4**	6*	2**,3**,4*
**Dizziness**	AUC (df = 3,30)	3.375	0.031	*	NS	NS	NS	NS	NS
	Peak (df = 3,30)	3.055	0.043	*	NS	NS	NS	NS	NS
	T_max_	13.13	0.004	NS	NS	NS	NS	NS	NS
	T-C (df = 3,270)	1.521	0.052						
**Confusion**	AUC (df = 3,30)	2.347	0.093						
	Peak (df = 3,30)	2.395	0.088						
	T_max_	8.10	0.044	NS	NS	NS	NS	NS	NS
	T-C (df = 3,270)	1.679	0.022	0.5*,0.75*,2**		0.5**,0.75**,1**, 1.5**,2*	2**		1.5*
**Changes in** **Distances**	AUC (df = 3,30)	2.071	0.125						
	Peak (df = 3,30)	1.530	0.226						
	T_max_	9.87	0.020	NS	NS	NS	NS	NS	NS
	T-C (df = 3,270)	1.513	0.054						
**Changes in** **Colors**	AUC (df = 3,30)	3.184	0.038	NS	NS	*	NS	NS	NS
	Peak (df = 3,30)	2.310	0.096						
	T_max_	6.38	0.095						
	T-C (df = 3,270)	1.352	0.120						
**Changes in lights**	AUC (df = 3,30)	2.880	0.052						
	Peak (df = 3,30)	2.652	0.067						
	T_max_	7.03	0.071						
	T-C (df = 3,270)	1.661	0.024		1*	0.5**,0.75**,1**, 1.5**,2**		0.5**,0.75**,1.5*	0.5**,0.75**,2*
**Changes in hearing**	AUC (df = 3,30)	1.102	0.364						
	Peak (df = 3,30)	1.574	0.216						
	T_max_	7.32	0.062						
	T-C (df = 3,270)	1.587	0.037		0.5**,0.75**,1*		0.5**,0.75**,1*	0.5*	
**Different body feeling**	AUC (df = 3,30)	4.905	0.007	NS	NS	**	NS	NS	NS
	Peak (df = 3,30)	7.766	0.001	NS	**	**	NS	NS	NS
	T_max_	14.43	0.002	NS	NS	*	NS	NS	NS
	T-C (df = 3,270)	3.535	<0.001		0.5**,0.75**,1**	0.5**,0.75**,1**, 1.5**,2**,3**	0.5**,0.75**,1**	1.5*,3**	0.5**,0.75**,1**,1.5**,3*
**Different surroundings**	AUC (df = 3,30)	1.426	0.255						
	Peak (df = 3,30)	2.147	0.115						
	T_max_	9.04	0.029	NS	NS	NS	NS	NS	NS
	T-C (df = 3,270)	1.243	0.195						
**ARCI questionnaire subscales**									
**ARCI-PCAG**	AUC(df = 3,30)	12.704	<0.001	**	NS	NS	**	NS	**
	Peak (df = 3,30)	6.992	0.001	**	NS	NS	*	NS	**
	T_max_	11.64	0.009	*	NS	NS	NS	NS	NS
	T-C (df = 3,150)	6.992	<0.001	1.5*,2**,3**,4**,6**		1.5**,2*,6**	1.5**,2**,3**,4**,6**	2**,3**,6**	1.5**,2**,3**,4**
**ARCI-MBG**	AUC (df = 3,30)	20.441	<0.001	NS	*	**	NS	**	**
	Peak (df = 3,30)	23.217	<0.001	NS	**	**	*	*	**
	T_max_	18.88	<0.001	*	*	*	NS	NS	NS
	T-C (df = 3,150)	11.162	<0.001		1.5**,2**	1.5**,2**,3**,4*	1.5**,2**	1.5**,2**,3**	1.5**,2**,3**
**ARCI-LSD**	AUC (df = 3,30)	3.787	0.020	NS	NS	*	NS	NS	NS
	Peak (df = 3,30)	3.290	0.034	NS	NS	NS	NS	NS	NS
	T_max_	15.43	0.001	*	NS	*	NS	NS	NS
	T-C (df = 3,150)	2.357	0.005/0.025	1.5**,2**,3**	1.5**	1.5**,2**,3*,4**	3*	4**	
**ARCI-BG**	AUC (df = 3,30)	13.389	<0.001	NS	NS	*	**	NS	**
	Peak (df = 3,30)	12.759	<0.001	NS	NS	**	**	NS	**
	T_max_	14.90	0.001	*	NS	*	NS	NS	NS
	T-C (df = 3,150)	5.124	<0.001	3**	1.5**	1.5**,2**,3**	1.5**,2**,3**,4*	2*,3**	1.5**,2**,3**,4**
**ARCI-A**	AUC (df = 3,30)	17.750	<0.001	NS	**	**	*	NS	**
	Peak (df = 3,30)	24.674	<0.001	NS	**	**	*	NS	**
	T_max_	17.60	0.001	NS	*	*	NS	NS	NS
	T-C (df = 3,150)	9.197	<0.001	1.5*	1.5**,2**,3*	1.5**,2**,3**,4**	1.5**,2**	2**,3**	1.5**,2**,3**,4**
**VESSPA questionnaire subscales**									
**VESSPA-S**	AUC (df = 3,30)	7.671	0.001	**	NS	NS	**	NS	NS
	Peak (df = 3,30)	9.517	<0.001	**	NS	*	*	NS	NS
	T_max_	21.03	<0.001	*	*	*	NS	NS	NS
	T-C (df = 3,150)	2.989	<0.001	1.5**, 2**,3**,4**,6**		6**	1.5**, 2**,3**,4*,6**	6**	1.5**,2**,3**
**VESSPA-ANX**	AUC (df = 3,30)	28.198	<0.001	NS	**	**	*	**	**
	Peak (df = 3,30)	31.959	<0.001	NS	**	**	**	NS	**
	T_max_	21.51	<0.001	*	*	*	NS	NS	NS
	Time Course (df = 3,150)	20.031	<0.001		1.5**,2**,3**	1.5**,2**,3**,4**,6**	1.5**,2**,3*	2**,3**,4**,6**	1.5**,2**,3**,4**,6**
**VESSPA-CP**	AUC (df = 3,30)	3.445	0.029	NS	NS	NS	NS	NS	NS
	Peak (df = 3,30)	3.604	0.025	NS	NS	NS	NS	NS	NS
	T_max_	8.88	0.031	NS	NS	NS	NS	NS	NS
	T-C (df = 3,150)	2.159	0.010	1.5**,2**,3*		1.5**,2**,3**	1.5**,2*,3*	1.5**,2**,3*	
**VESSPA-SOC**	AUC (df = 3,30)	11.817	<0.001	NS	**	**	NS	NS	**
	Peak (df = 3,30)	13.779	<0.001	NS	**	**	NS	NS	**
	T_max_	16.53	0.001	NS	NS	NS	NS	NS	NS
	T-C (df = 3,150)	8.619	<0.001	1.5**,2*	1.5**,2**	1.5**,2**,3**,4*	1.5**	1.5**,2**,3**	1.5**,2**,3**
**VESSPA-ACT**	AUC (df = 3,30)	15.151	<0.001	NS	NS	**	NS	**	**
	Peak (df = 3,30)	20.330	<0.001	NS	**	**	NS	NS	**
	T_max_	15.03	0.002	*	NS	NS	NS	NS	NS
	T-C (df = 3,150)	10.297	<0.001	1.5**	1.5**,2**	1.5**,2**,3**,4*	1.5**	1.5**,2**,3**	1.5**,2**,3**
**VESSPA-PS**	AUC (df = 3,30)	3.463	0.028	NS	NS	*	NS	NS	NS
	Peak (df = 3,30)	5.497	0.004	NS	NS	**	NS	NS	*
	T_max_	13.53	0.004	NS	NS	NS	NS	NS	NS
	T-C (df = 3,150)	4.025	<0.001		1.5**	1.5**,2**,3*,4*		2**,4*	1.5**,2**
**Cortisol**	AUC (df = 3,30)	19.266	<0.001	NS	**	**	**	NS	**
	Peak (df = 3,30)	17.240	<0.001	NS	**	**	**	NS	**
	T_max_	17.340	0.001	NS	NS	NS	NS	NS	NS
	T-C (df = 3,120)	8.404	<0.001	NS	1**,2**	1**,2**	1**,2**	NS	1**,2**

Peak = peak effects from 0 to 6 hours measured by mmHg (systolic blood pressure [SBP]; diastolic blood pressure [DBP]), bpm (heart rate [HR]),

°C [temperature (T)], mm [pupil diameter (PD); mm visual analog scale (VAS0)], and score [Addiction Research Center Inventory (ARCI), score Evaluation of Subjective Effects of Substances with Abuse Potential questionnaire (VESSPA-SEE)] and expressed as mean.

AUC = area under the curve from 0 to 6 h measured by units x hours and expressed as mean.

Tmax = time to reach peak effects measured by hours and expressed as median.

df = degree of freedom.

For Peak, AUC and T-C an ANOVA and multiple Tukey post hoc comparisons were used. Statistical differences among conditions are presented as **p < 0.01 and *p < 0.05.

For Tmax a Friedman test and multiple Wilcoxon post-hoc comparisons were used. Differences among conditions are presented as *p < 0.008; **p < 0.002.

**Table 2:** Summary of Peak effects (maximal effect, mean value and standard error) for physiological, subjective and cortisol concentrations (Cmax) (n = 11).

	Placebo	Alcohol	Mephedrone	Mephedrone + alcohol
**Physiological effects**				
SBP	-5.82 ± 2.96	-5.00 ± 4.52	35.45 ± 4.28	40.36 ± 3.57
DBP	0.27 ± 3.10	-1.73 ± 3.86	19.64 ± 2.08	22.45 ± 1.83
HR	-7.18 ± 2.28	9.91 ± 4.35	32.27 ± 6.03	40.09 ± 5.25
T	-0.94 ± 0.12	-0.47 ± 0.14	-0.56 ± 0.21	-0.56 ± 0.16
PD	0.00 ± 0.00	0.00 ± 0.00	1.64 ± 0.35	1.91 ± 0.33
Maddox-wing	0.00 ± 0.27	1.41 ± 0.39	-3.45 ± 0.82	-1.41 ± 0.88
**Visual Analogue Scales**				
High	0.00 ± 0.00	1.55 ± 1.55	47.36 ± 9.31	55.09 ± 7.90
Drunkenness	0.00 ± 0.00	49.36 ± 7.75	0.00 ± 0.00	34.18 ± 6.49
Stimulated	0.00 ± 0.00	23.00 ± 5.77	42.73 ± 8.56	53.18 ± 8.17
Any effect	0.00 ± 0.00	49.91 ± 7.40	51.18 ± 9.27	56.09 ± 7.68
Good effects	0.00 ± 0.00	23.18 ± 5.20	51.09 ± 8.59	63.36 ± 8.49
Bad effects	0.00 ± 0.00	14.18 ± 7.26	3.45 ± 2.06	4.55 ± 2.05
Liking	0.00 ± 0.00	17.18 ± 7.19	44.09 ± 8.59	50.27 ± 8.31
Content	0.00 ± 0.00	30.91 ± 6.73	43.64 ± 8.24	56.09 ± 8.16
Drowsiness	8.45 ± 2.62	29.09 ± 8.14	12.36 ± 4.81	17.00 ± 3.95
Dizziness	0.00 ± 0.00	20.27 ± 8.07	6.18 ± 5.70	11.36 ± 8.00
Confusion	0.00 ± 0.00	20.64 ± 7.67	11.00 ± 5.85	15.73 ± 9.29
Changes in Distances	0.00 ± 0.00	11.55 ± 6.14	11.64 ± 6.53	13.09 ± 6.78
Changes in Colors	0.00 ± 0.00	4.73 ± 2.46	6.27 ± 3.45	8.55 ± 3.84
Changes in lights	0.00 ± 0.00	6.36 ± 2.76	6.64 ± 4.11	11.64 ± 5.73
Changes in hearing	0.00 ± 0.00	3.18 ± 1.58	14.18 ± 9.29	6.27 ± 4.13
Different body feeling	0.00 ± 0.00	13.27 ± 5.21	30.91 ± 9.01	33.18 ± 9.67
Different surroundings	0.00 ± 0.00	9.91 ± 5.39	10.73 ± 3.61	15.36 ± 7.80
**ARCI questionnaire subscales**				
ARCI-PCAG	1.00 ± 0.65	6.18 ± 1.09	2.82 ± 1.19	1.73 ± 1.58
ARCI-MBG	0.09 ± 0.09	2.27 ± 0.49	6.45 ± 1.75	10.36 ± 1.18
ARCI-LSD	-0.45 ± 0.25	1.55 ± 0.85	0.73 ± 0.74	1.55 ± 0.96
ARCI-BG	0.00 ± 0.23	-2.27 ± 0.54	2.64 ± 0.89	3.82 ± 1.06
ARCI-A	0.27 ± 0.14	2.00 ± 0.47	4.82 ± 0.84	6.55 ± 0.82
**VESSPA questionnaire subscales**				
VESSPA-S	1.09 ± 1.09	9.36 ± 2.49	4.27 ± 1.47	6.09 ± 1.40
VESSPA-ANX	0.00 ± 0.00	1.55 ± 0.41	9.00 ± 1.72	12.36 ± 1.46
VESSPA-CP	0.00 ± 0.00	1.64 ± 0.65	0.45 ± 0.25	1.91 ± 0.91
VESSPA-SOC	0.09 ± 0.09	4.09 ± 1.46	7.36 ± 2.22	13.00 ± 1.73
VESSPA-ACT	0.27 ± 0.19	4.36 ± 0.96	8.18 ± 2.14	12.55 ± 1.52
VESSPA-PS	0.00 ± 0.00	0.64 ± 0.39	1.27 ± 0.45	2.45 ± 0.87
**Cortisol**	14.93 ± 4.50	16.20 ± 4.88	22.23 ± 6.70	23.85 ± 7.19

SBP: systolic blood pressure, mm; DBP: diastolic blood pressure, mmHg; HR: heart rate, beats per minute; T: temperature, °C; PD: pupil diameter, mm; visual analogue scale, mm, ARCI: Addiction Research Center Inventory [ARCI], score; VESSPA: Evaluation of Subjective Effects of Substances with Abuse Potential questionnaire, score.

Only statistically significant results are showed (see **Table 1** for comparisons). Conditions are mephedrone plus alcohol (mephedrone–alcohol), mephedrone plus placebo alcohol (mephedrone), placebo mephedrone plus alcohol (alcohol), and placebo mephedrone plus placebo alcohol (placebo).

**Figure 1:**
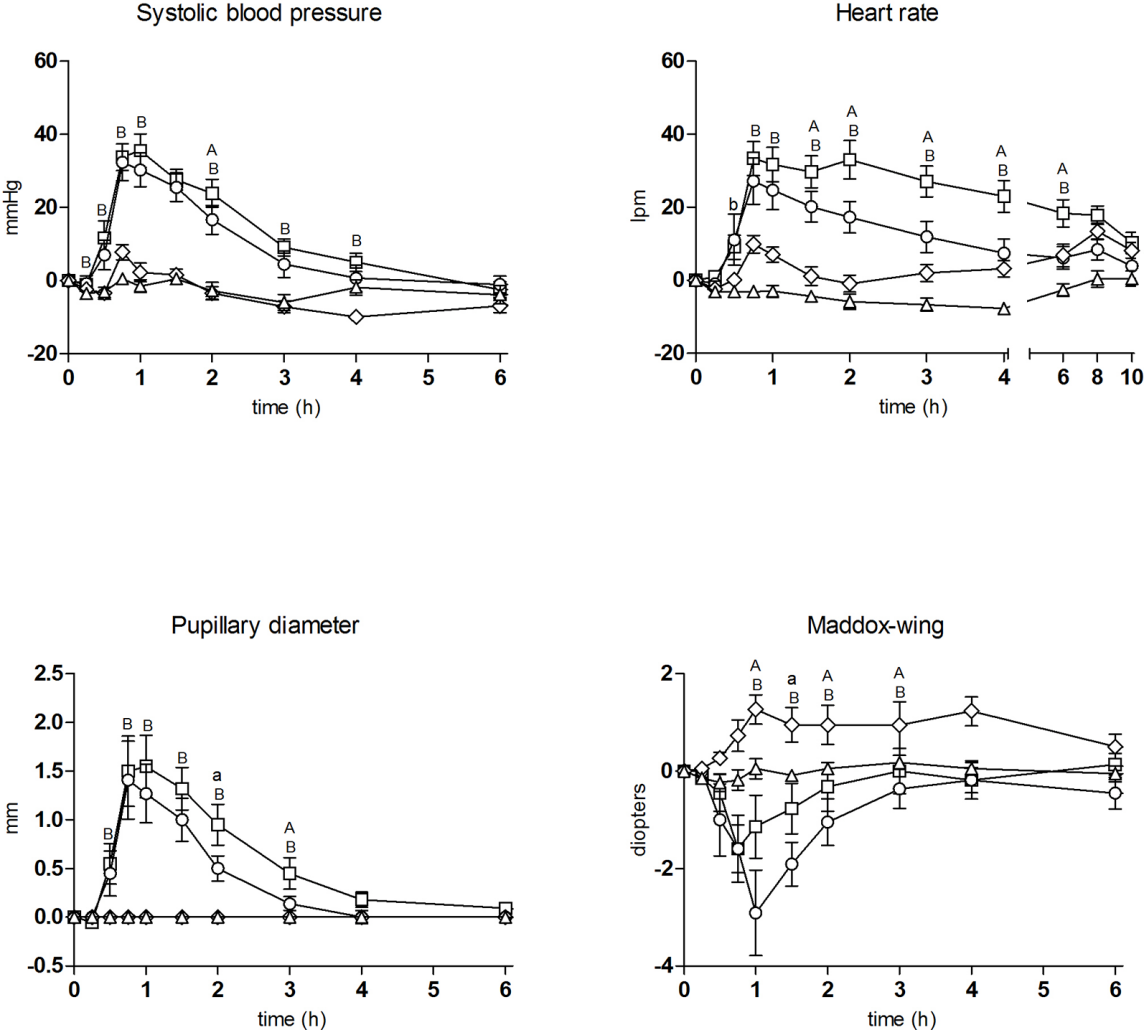
Time course of drug effects (n = 11, mean, standard error) on physiological and psychomotor performance (differences from baseline). □ mephedrone + alcohol; ○ mephedrone; ◊ alcohol; Δ placebo; Significant differences between mephedrone vs mephedrone + alcohol (a: p < 0.05/A: p < 0.01); alcohol vs mephedrone + alcohol (b: p < 0.05/B: p < 0.01).

**Figure 2:**
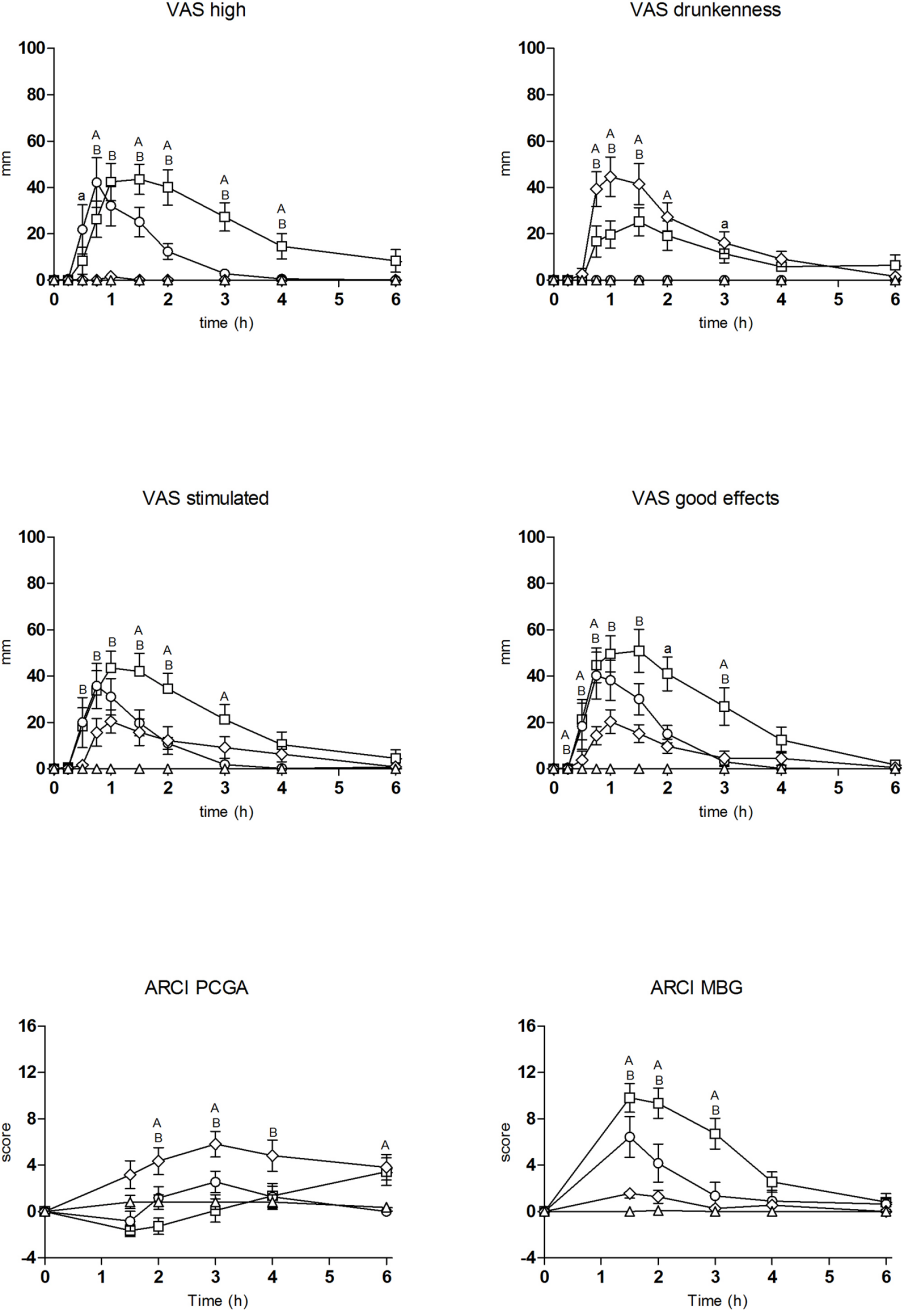
Time course of drug effects (n = 11, mean, standard error) on subjective effects (differences from baseline). □ mephedrone + alcohol; ○ mephedrone; ◊ alcohol; Δ placebo; Significant differences between mephedrone vs mephedrone + alcohol (a: p < 0.05/A: p < 0.01); alcohol vs mephedrone + alcohol (b: p < 0.05/B: p < 0.01).

**Figure 3:**
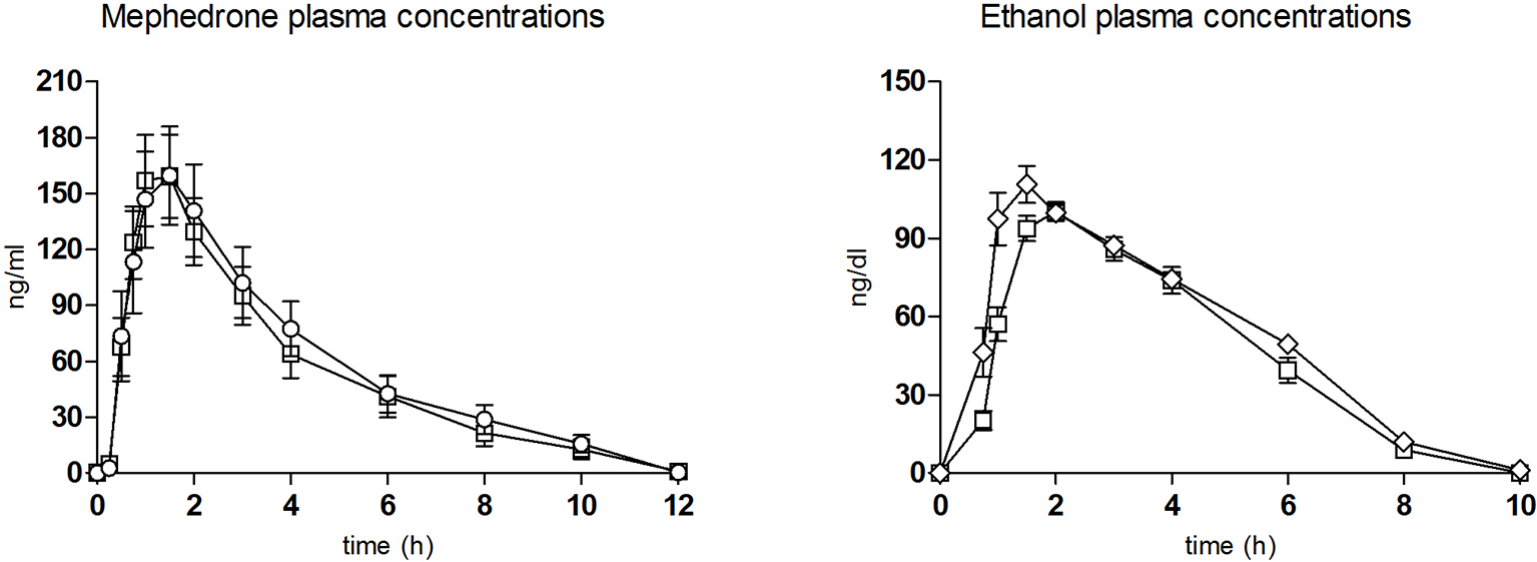
Plasma concentration over time curves of mephedrone (left) and ethanol (right) (n = 11, mean, standard error). ○ mephedrone; ◊ alcohol; □ mephedrone + alcohol.

The T-C (differences from baseline) for the most relevant physiological, psychomotor, and subjective effects are shown in **Figures 1** and **2**, respectively. Concentrations over time and pharmacokinetic parameters of mephedrone and alcohol in plasma are presented in **Figure 3** and **Table 3**, respectively. The effects of all drug/experimental conditions on plasma cortisol levels are depicted in **Tables 1** and **2**.

**Table 3:** Pharmacokinetics parameters of mephedrone and alcohol in plasma (n = 11).

Pharmacokinetic parameters	Cmax (ng/ml)	AUC_0-6_ (ng/ml h^-1^)	AUC_0-24_ (ng/ml h^-1^)	Tmax (h)	Ke (h^-1^)	t1/2 (h)
Mephedrone						
Mephedrone and ethanol	175.7 ± 71.1	516.8 ± 264.6	709.8 ± 477.1	1.5 (0.75–2)	0.35 ± 0.14	2.32 ± 1.01
Mephedrone	172.6 ± 82.9	549.0 ± 315.0	778.4 ± 512.9	1.5 (0.5–2)	0.29 ± 0.09	2.68 ± 0.92
p-value	NS	NS	NS	NS	NS	NS
						
	**Cmax (ng/ml)**	**AUC_0-6_ (ng/ml h^-1^)**	**AUC_0-10_ (ng/ml h^-1^)**	**Tmax (h)**		
Ethanol						
Mephedrone and ethanol	103.8 ± 14.1	389.8 ± 73.3	447.0 ± 96.8	2.0 (1.5–2)		
Ethanol	121.1 ± 14.9	438.4 ± 36.8	512.9 ± 45.2	1.5 (1–2)		
p-value	0.030	0.048	0.020	0.014		

AUC: area under the curve. SD: standard deviation. T_max_ is shown as median (range) values. NS: not statistically significant. A value of p < 0.05 was considered statistically significant.

No serious adverse events were observed. No hallucinations, psychotic episodes, or any other psychiatric symptoms were experienced during the sessions. None of the participants required specific therapy or special care during the study. All 11 subjects completed the study.

### Physiological Effects

Regarding physiological effects, the two conditions including mephedrone (combination condition and mephedrone condition) produced an increase in SBP, DBP, HR, and PD as compared with the placebo condition (when considering both peak effects and AUC). The more relevant differences for HR increase appeared between the combination and mephedrone conditions. The former produced an increase of 40 ± 17 beats per min (bpm) at 1 h following the first drug administration whilst for the latter the peak effects was 32 ± 20 bpm at 0.75 h. In statistical terms, only significant differences were detected for AUC_0-6_ (142.33 _mm_ × h for the combination condition, and 70.91 mm × h for the mephedrone one) and T-C points (1.5–6 h) for both. For the remaining physiological variables (SBP, DBP, T, and PD), only significant differences in several T-C points were observed between the combination and mephedrone conditions (no AUC or peak effects).

The alcohol condition produced very slight effects on SBP, DBP, HR, and T and none on DP compared with the placebo condition (non-statistically significant). Comparing the combination and alcohol condition, the mephedrone-alcohol effects on SBP, DBP, HR and DP were significantly higher than those produced by alcohol alone (when considering both peak effects and AUC) with significant differences at several T-C points.

In the Maddox-wing device, the combination and mephedrone conditions led to an increase in the degree of esophoria compared with placebo (when considering peak effects and/or AUC). Esophoria induced by the combination condition was approximately two to three-fold lower (-1.41 ± 2.91 diopters) in comparison with the mephedrone one (-3.45 ± 2.73 diopters) with only significant differences in several T-C points (1–1.5 h). Alcohol slightly increased exophoria (+1.41 ± 1.30 diopters), relaxation of ocular musculature, but only significant differences in several T-C points were obtained when compared with the placebo. The combination condition scored approximately midway between the mephedrone and alcohol conditions.

### Subjective Effects

The two conditions containing mephedrone caused an increase in subjective effects (VAS, ARCI, and VESSPA-SEE) compared with placebo (**Tables 1** and **2**).

The combination and mephedrone conditions led to significant changes in the ratings of “high,” “stimulated,” “any effect,” “good effects,” “liking,” “content” and also in “change in distances and different, changed or unreal body feeling” in comparison with placebo. In general terms, the combination condition produced higher and more prolonged scores with statistically significant differences in AUC and several T-C points compared with mephedrone alone. In addition, for “stimulated,” “any effect,” “good effects,” “liking,” and “content” scores these differences were statistically significant for peak effects and, in the case of “high,” also for T_max_. As expected, statistically significant differences were detected in drunkenness rating scores between conditions (AUC, peak, T_max_, and T-C, **Tables 1** and **2**).

The alcohol condition produced an increase in the ratings of “drunkenness,” “content,” “stimulated,” “any effect,” “good effects,” and “liking” compared with placebo (**Table 2**).

Regarding “drunkenness,” the most characteristic effect for alcohol, a no significant lower score was observed for the combination condition in comparison to the alcohol condition (peak effect 34.18 ± 21.53 vs 49.36 ± 25.70 mm, respectively; **Table 2**), but significant differences, however, were only found at several T-C points (0.75–1.5 h). In contrast, significant differences were detected for “content” effects in AUC, peak effects, and T-C points (0.5–3 h) with higher scores in the combination condition compared with the alcohol one (**Tables 1** and **2**).

In the ARCI questionnaire, both conditions including mephedrone produced an increase in the scores of MBG (euphoria), BG (intellectual efficiency and energy) (**Tables 1** and **2**), A (amphetamine-like effects), and LSD (dysphoria) subscales in comparison to placebo (when considering AUC, peak effects, and both). The MBG, BG, and A peak scores were 10.36 ± 3.91, 3.82 ± 3.52, and 6.55 ± 2.73 points for the combination condition and 6.45 ± 5.80, 2.64 ± 2.94, and 4.82 ± 2.79 points for the mephedrone condition, respectively. For MBG, significant differences were detected in AUC, peak effects, and several T-C points (1.5–3h) between conditions. For the other subscales only significant differences were detected at several T-C points. Conversely, for the LSD subscale no significant differences were detected between both conditions.

Alcohol produced a statistically significant increase in the PCAG-sedation subscale in comparison to placebo. Compared with the alcohol condition, the combination with mephedrone reduced the sedation induced by alcohol (peak effect 6.18 ± 3.60 vs 5.26 ± 1.58) with statistical differences in peak effects and AUC that remained statistically significant during 2.5 h in the T-C analysis (1.5–4 h). In contrast, MBG peak difference scores were lower for alcohol compared to the combination condition (2.27 ± 1.62 vs 10.36 ± 3.91, respectively) with statistical differences in peak effects, AUC, and T-C analysis (1.5–3 h) (**Tables 1** and **2**).

Regarding the VESSPA-SEE questionnaire, the combination and mephedrone conditions increased all the subscales compared with placebo. The combination condition, in comparison to the mephedrone one, presented statistical differences in peak effects for ACT (activity and energy) and ANX (psychosomatic anxiety) subscales and in several T-C points for all the subscales (**Tables 1** and **2**). With respect to the alcohol condition, statistical differences in peak effects, AUC, and T-C points for ANX, SOC, (pleasure and sociability) and ACT subscales and only in peak effects for CP subscale were detected compared to the combination one.

In the pharmacologic drug class identification questionnaire, the combination condition (mephedrone plus alcohol) was correctly identified by all subjects (100% designer drug and 100% alcohol). For the mephedrone condition (mephedrone plus placebo), mephedrone was identified by eight subjects as a designer drug (72.7%), as a stimulant (18.2%) by two, and as placebo (9.1%) by one, whilst placebo was identified correctly by all the subjects (100%). With respect to the alcohol/placebo alcohol conditions, the placebo was identified as such by nine subjects (81.8%), and as a designer drug (9.1%) and cannabinoids (9.1%) by one subject each, whilst alcohol was identified correctly by all subjects (100%).

All the active conditions (mephedrone, alcohol, and the combination) were well tolerated and no mania, hallucinations or psychotic reactions were observed or reported during the study.

### Pharmacokinetic Measures

#### Mephedrone and Alcohol Concentrations

The pharmacokinetic parameters for mephedrone and alcohol are summarized in **Table 3** and **Figure 3**.

When the two conditions containing mephedrone were compared, no significant differences were found in the pharmacokinetic parameters (**Table 3**). In the combination condition, mephedrone concentrations peaked at 1.5 h. In the mephedrone condition, mephedrone concentrations peaked at 1.5 h. In both conditions, at 10 h following first drug administration, mephedrone concentrations declined to mean values of 15.73 and 12.76 ng/ml until undetectable levels at 24 h, respectively (**Figure 3**).

Regarding alcohol, significant differences in pharmacokinetics were detected. Alcohol concentrations peaked at 2 h (in the combination condition and at 1.5 h in the alcohol one. Significant differences in AUC were detected between alcohol and combination conditions.

#### Cortisol Concentrations

Kinetic parameters for cortisol are summarized in **Tables 1** and **2**. Plasma cortisol concentrations were significantly higher (peak and AUC) after the administration of the combination and mephedrone conditions as compared with placebo.

Cortisol concentrations peaked at 2 h with a mean peak of 23.85 ± 1.31 µg/dl after the combination condition and 22.23 ± 1.23 µg/dl after mephedrone administration. The combination condition also showed significant differences in comparison to alcohol, cortisol concentrations peaked at 2 h with a mean peak of 16.20 ± 0.82 µg/dl. AUC for combination, mephedrone and alcohol conditions were 96.05, 87.29 and 62.31 ng/ml·h^1^, respectively.

Significant differences in peak, AUC and several T-C points (1 and 2 h) were observed between the combination and mephedrone conditions in comparison to placebo, and also between alcohol and the combination conditions in comparison to mephedrone.

## Discussion

To the best of our knowledge, this study provides the first data in humans about the pharmacodynamics and pharmacokinetics of mephedrone and alcohol interactions and completes previous results on neurocognitive performance effects (de Sousa Fernandes Perna et al., 2016). Our findings demonstrate the increased pharmacological effects of the co-administration of mephedrone and alcohol compared to single drug administration.

The 200 mg oral administration of mephedrone reproduced pharmacological effects which concurred with the sole experimental study performed to date in humans (Papaseit et al, 2016). Our results demonstrate that mephedrone produced a significant increase in BP, HR, and PD. It also induced stimulant-like effects (euphoria, well-being, feelings of pleasure) and mild changes in perceptions. All these physiological and subjective effects were of rapid onset and short duration. The increase in VAS scores (stimulated, high, good effects, liking) and ARCI (subscales MBG, BG, and A) caused by mephedrone were within the range of previous studies with psychostimulant drugs with a well-known abuse potential (Farré et al., 1993; Hernández-López et al., 2002). Moreover, its faster and shorter duration confirmed results obtained in the previous human investigation (Papaseit et al., 2016). The administration of an oral dose of 0.8 g/kg of alcohol replicated the typical effects of acute alcohol intoxication characterized by an increase in the ratings of drunkenness, sedation, and mild effects on physiological responses compared with placebo.

The co-administration of mephedrone and alcohol amplified cardiovascular effects, producing a more marked increase in HR in comparison with mephedrone alone. In turn, these results are consistent with a previous description of cardiovascular toxicity associated with mephedrone and alcohol co-ingestion (McGaw and Kankam, 2010). Similar findings have also been observed after the concomitant administration of other psychostimulants drugs (MDMA, methamphetamine, cocaine) in combination with alcohol in healthy volunteers (Pérez-Reyes and Jeffcoat, 1992; Farré et al., 1993; Higgins et al., 1993; McCance-Katz et al., 1993; Mendelson et al., 1995; Hernández-López et al., 2002; Dumont et al., 2010).

In addition, the combination of mephedrone and alcohol produced mydriasis and esophoria, an indicator of extraocular muscle tension, two specific acute psychostimulant-like effects, although slighter in comparison to mephedrone alone. These results are in line with those observed after other psycho-stimulant drugs and alcohol co-administration (Mas et al., 1999; de la Torre et al., 2000; Farré et al., 2015). Alcohol, as expected from the extrapolation of results obtained from other psychostimulant-alcohol interaction studies, attenuated the rise in PD and extraocular musculature contraction induced by mephedrone. Furthermore, the addition of alcohol to mephedrone increased the maximal psychostimulant effects, maintaining higher measures for euphoria and well-being for a longer period of time in comparison to mephedrone alone, which maximal effects are faster (Papaseit et al., 2016). Among conditions, the most remarkable difference was in the mephedrone-alcohol combination. It produced during 4 h relevant increases in subjective scores which were intense during the three first hours compared with 1–2 h following mephedrone alone.

Alcohol administration, equivalent four to six alcoholic beverages, in combination with mephedrone resulted in decreased drunkenness and reduced sedative effects producing mixed scores (ARCI-PCAG and VAS drowsiness) between the mephedrone and alcohol alone conditions. Mephedrone moderated the effects induced by alcohol in a similar manner to that observed after MDMA experimental administration (Hernández-López et al., 2002).

Overall, the combination of mephedrone and alcohol slightly delayed peak effects and increased maximal effects which remained high with no changes in their total duration. Cardiovascular and subjective effects after mephedrone-alcohol co-administration started at 0.25–5 h, peaked at approximately 0.75–1.5 h after administration, and returned to pre-dose values at 4–8 h after administration with approximately 3–4 h of marked effects. In general terms, mephedrone induced lower effects than the combination and almost overlapped with our own observations in a previous study in which the same dose of mephedrone was administered (Papaseit et al., 2016). Mephedrone effects were observed between 0.5-1 h and most returned to baseline 2–3 h after drug administration, but some last more than 3 h (HR, temperature, stimulated or any effect).

With respect to the pharmacokinetics of the mephedrone-alcohol combination, the most relevant finding was that alcohol did not modify the plasma levels of mephedrone. The maximal concentrations of mephedrone after the combination administration were within the range of those obtained after mephedrone alone and concurred with previously published data (Papaseit et al., 2016). This finding suggests that the pharmacokinetics of mephedrone is not altered when alcohol is concurrently administered.

With reference to alcohol pharmacokinetics, statistically significant differences were detected in C_max_, AUC, and T_max_. In this respect, other psychostimulant-alcohol studies have also reported slightly lower alcohol C_max_ in combination conditions related to changes in alcohol metabolism and absorption rate (Farré et al., 1993; McCance-Katz et al., 1993). In the case of mephedrone, initial results suggest a similar kinetic scenario.

An adequate pharmacological effect in relation to pharmacokinetics was thus observed despite the biological variability among subjects. Our results suggest that the higher abuse liability exhibited for mephedrone when concomitantly consumed with alcohol can be attributed to the early onset and maintenance of the subjective/pleasant effects in comparison to mephedrone alone. It should be emphasized that the short mephedrone half-life and T_max_ following its co-administration with alcohol could partially explain the mephedrone-alcohol binge pattern among regular users.

Higher increases in cortisol plasma concentrations were found after the mephedrone-alcohol co-administration and mephedrone alone in comparison to the rest of conditions. This pattern of response has already been observed after cocaine and alcohol (Farré et al., 1997) and MDMA and alcohol administration (Hernández-López et al., 2002). Interestingly, both combinations with alcohol, and also MDMA and cocaine alone, produced significant cortisol increases (Farré et al., 1997; Harris et al., 2002; Hernández-López et al., 2002; Kuypers et al., 2013; Kuypers et al., 2015). As far as we know, the precise mechanism of this effect is poorly understood. Although the role of cortisol in the acute effects of mephedrone has as yet to be described, it could be extrapolated to MDMA and related-amphetamines (Mas et al., 1999). The serotonergic effects of mephedrone might stimulate the hypothalamo-pituitary-adrenal axis, leading to an increase in cortisol plasma concentrations as previously described by other psychostimulants as MDMA (Seibert et al., 2014; Papaseit et al., 2016; Strajhar et al., 2019).

Integrating the results of the present study and the previous one on psychomotor performance (de Sousa Fernandes Perna et al., 2016), it seems that mephedrone reduced some of the subjective feelings of sedation induced by alcohol, but its stimulatory effects were not enough to compensate for the impairing effects of alcohol on most performance parameters. This dissociation between subjective and objective sedation measures is of interest. Subjects may feel less sedated by alcohol and psychomotor abilities remain impaired or unchanged. The potential impact of this dissociation in terms of driving safety is unknown, but it may be plausible that subjects would consider they are driving better when actual performance continues to be impaired by the effect of alcohol. Similar dissociation has been reported when other psychostimulants (e.g. cocaine, MDMA) have been administered simultaneously with alcohol (Farré et al., 1993; Farré et al., 1997; Hernández-López et al., 2002).

The present study has several limitations which are mainly associated with its experimental design. Firstly, the moderate sample size. Secondly, a non-representative sample that not allows to generalize the results across gender (absence of women due to unknown and potential seriously fetal risk). Thirdly, the evaluation of only one dose level of mephedrone and alcohol (users usually are subjected to repeated drug consumption in a single session). Fourthly, the alcohol blind was potentially insufficient. Nonetheless, we conducted a placebo controlled study, randomized, within-subjects, various control conditions (combination condition, mephedrone condition, alcohol condition, and placebo condition). The results provide novel data on pharmacodynamics and pharmacokinetics of mephedrone-alcohol combination.

In summary, the concomitant administration of mephedrone and alcohol produced a significant increase in cardiovascular effects and induced more intense and prolonged feelings of euphoria and well-being, in comparison to mephedrone alone. Mephedrone reduced the drunkenness and sedation produced by alcohol. These effects could encourage the consumption of larger amounts of mephedrone and alcohol, placing the recreational user at a heightened risk for potentially toxic effects. The results presented are like those obtained with the combination of alcohol and other psychostimulants, suggesting that the abuse liability of the simultaneous consumption is greater than that induced by mephedrone alone.

## Data Availability Statement

The datasets generated for this study are available on request to the senior author (Magi Farré, magi.farre@uab.cat).

## Ethics Statement

The protocol was approved by the local Research Ethics Committee (CEIC-Parc de Salut Mar, Barcelona, Spain). The study was conducted in accordance with the Declaration of Helsinki and Spanish laws concerning clinical trials and registered in ClinicalTrials.gov (number NCT02294266).

## Author Contributions

MF, EP, CP-M, ES, RT, KK, ET, and JR conceptualized the study design. EP, CP-M, JM, ES, FF, MT, and MF collected the data. RT and EO analyzed mephedrone and alcohol concentrations. EP, CP-M, EO, and MF analyzed the data. EP, MF, CP-M, MT, FF, RT, ES, KK, ET, and JR wrote and reviewed the manuscript.

## Funding

This work was supported in part by grants from Instituto de Salud Carlos III (ISCIII, FIS-FEDER, FIS PI11/01961, FISPI17/01962), ISCIII-Red de Trastornos Adictivos (RTA RD16/0017/0003 and RD16/0017/0010) and The European Commission [HOME/2014/JDRU/AG/DRUG/7082, Predicting Risk of Emerging drugs with In silico and Clinical Toxicology (PREDICT)]. EP has a Juan Rodes fellowship (ISC-III, JR16/00020). We are grateful to Esther Menoyo (RN), Marta Pérez (RN), Soraya Martín (RN), Clara Gibert (RN), and Joan Mestres (PsyD) for their valuable assistance throughout the clinical study.

## Conflict of Interest

The authors declare that the research was conducted in the absence of any commercial or financial relationships that could be construed as a potential conflict of interest.

The reviewer ML declared a past co-authorship with several of the authors KP, JR to the handling editor.
